# Multiscale and hierarchical wrinkle enhanced graphene/Ecoflex sensors integrated with human-machine interfaces and cloud-platform

**DOI:** 10.1038/s41528-022-00189-1

**Published:** 2022-07-05

**Authors:** Jian Zhou, Xinxin Long, Jian Huang, Caixuan Jiang, Fengling Zhuo, Chen Guo, Honglang Li, YongQing Fu, Huigao Duan

**Affiliations:** 1 College of Mechanical and Vehicle Engineering, Hunan University, Changsha, 410082 China; 2 National Center for Nanoscience and Technology, Beijing, 100190 China; 3 Faculty of Engineering and Environment, Northumbria University, Newcastle upon Tyne, NE1 8ST United Kingdom; 4 Greater Bay Area Institute for Innovation, Hunan University, Guangzhou, 511300 Guangdong Province China

**Keywords:** Chemical engineering, Sensors

## Abstract

Current state-of-the-art stretchable/flexible sensors have received stringent demands on sensitivity, flexibility, linearity, and wide-range measurement capability. Herein, we report a methodology of strain sensors based on graphene/Ecoflex composites by modulating multiscale/hierarchical wrinkles on flexible substrates. The sensor shows an ultra-high sensitivity with a gauge factor of 1078.1, a stretchability of 650%, a response time of ~140 ms, and a superior cycling durability. It can detect wide-range physiological signals including vigorous body motions, pulse monitoring and speech recognition, and be used for monitoring of human respirations in real-time using a cloud platform, showing a great potential for the healthcare internet of things. Complex gestures/sign languages can be precisely detected. Human-machine interface is demonstrated by using a sensor-integrated glove to remotely control an external manipulator to remotely defuse a bomb. This study provides strategies for real-time/long-range medical diagnosis and remote assistance to perform dangerous tasks in industry and military fields.

## Introduction

Flexible, stretchable and wearable strain sensors have received extensive attention recently because large strains can be applied on the flexible/bendable/stretchable or curved substrates. They effectively change complex mechanical deformations into electrical signals, promising for applications in human health-monitoring systems, wearable internet of things (WIoT), human–machine interaction and soft robotics, etc^1–5^. These stretchable/flexible/wearable strain sensors can withstand a much larger strain (up to 500 %) and significant deformation when compared with their rigid counterparts (normally with a strain smaller than 5%)^6^. Therefore, they are explored for in-situ and precise measurements on the complex-shaped surfaces. Various types of stretchable strain sensors are currently available, for examples, capacitive sensors^7,8^, resistive sensors^9,10^, triboelectric sensors^11,12^, and piezoelectric sensors^13,14^. Among them, the resistive strain ones have been widely used for wearable sensing applications, because they not only have simple structures, convenient read-out circuit and low-cost microfabrication processes, but also offer good stretchability, high sensitivity and flexibility^15,16^.

Recently the relevant research has been focused on conductive materials integrated with flexible polymers to fabricate strain sensors. Various materials such as carbon blacks (CB), nanoparticles (NPs), carbon nanotubes (CNTs), graphene, nanowires (NWs), and hybrid micro/nanostructures have been applied for this purpose^17–23^. Among them, low-dimensional carbons (including CB, CNTs and graphene) are very attractive due to their good flexibility, large surface/volume ratio, good chemical and thermal stabilities, and good electrical conductivity^24–26^.

In order to realize their practical applications, these sensors should possess both high sensitivity and large stretchability. However, their sensitivity and stretchability are often considered contradictory between each other. Therefore, many researchers proposed various surface and interface engineering methods (such as fish-scale microstructures^27^, reversible microcrack formation^28^, microprism-array architecture^29^, acid-interface engineering^30^ and wrinkle structures, etc^31–33^.) to solve this dilemma for achieving high sensitivities and wide ranges of strain detection. Among these methods, application of wrinkle structures is one of the prevalent strategies. Previously Pegan et al. proposed a wrinkled platinum strain sensor, and achieved a tensile strain of 185% and a gauge factor (GF) of 42^34^. Based on a multiscale structural design, Xue et al. proposed a strain sensor using graphene and nanocrystalline carbon film with wrinkled structures, demonstrating an ultrahigh GF of 1071^35^. Chu et al. fabricated a wrinkle-based sensor using a pre-stretching method, and demonstrated a strain up to 300%^36^. Nevertheless, these developed strain sensors are still difficult to achieve a combined high sensitivity (GF > 1000) and high stretchability (>500%), as well as ultrafast responses and capabilities for a wide range of strain detections. It is critical to explore different strategies to overcome such limitations and develop strain sensors which are highly sensitive and stretchable, with good stability and ultra-fast responses, as well as a wide-range strain detection.

**Fig. 1:**
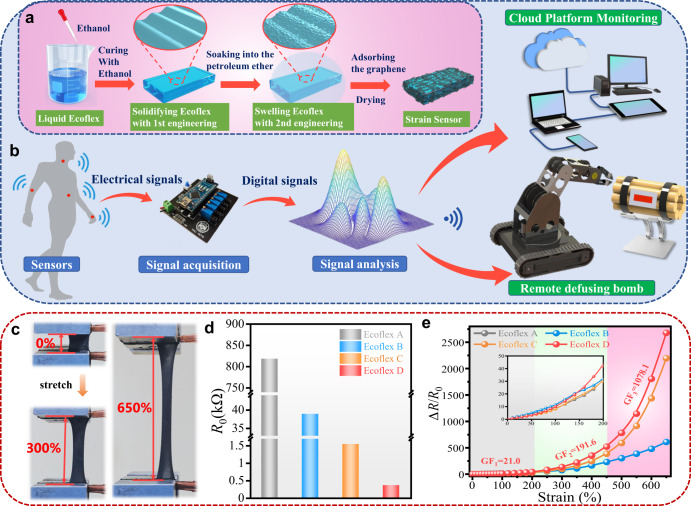
Fabrication processes, performance and application scenario of the graphene/Ecoflex strain sensor. **a** Schematic illustrations of fabrication processes for the graphene/wrinkled Ecoflex strain sensors; **b** Diagrams of the flexible sensors that can detect and capture the physiological signals from human body, signal acquisition unit, signal analysis unit, cloud platform monitoring and remote defusing bomb; **c** Photographs of the graphene/Ecoflex strain sensor under the tension strain of 0%, 300 and 650%; **d** The initial resistance of four types of graphene/Ecoflex strain sensor; **e** the relative resistance changes (∆*R*/*R*_0_) versus strain for four types of graphene/Ecoflex strain sensor.

In this study, an interface engineering strategy is proposed to design and fabricate flexible graphene/Ecoflex composite strain sensors by applying wrinkling mechanisms onto the Ecoflex substrate. We have applied a cost-effective and two-step method for the surface treatment of Ecoflex substrate (e.g., ethanol-assisted solidification and then immersion into petroleum ether) to form micro- and nanoscale wrinkle patterns, as shown in Fig. 1a. This can substantially increase the specific surface areas and interfacial bonding between Ecoflex and graphene, thus significantly improving its strain sensitivity. The fabricated flexible/stretchable strain sensor exhibits a high sensitivity with a GF of 1078.1, a large stretchability with a strain up to 650%, a good cycling durability (e.g., 3000 cycles with a strain of 200%), and a fast response time of ~140 ms. The sensor is capable of detecting and capturing wide-range physiological signals from the sound of speech and pulse vibration, to vigorous body motions. It can also be applied for real-time cloud-platform and multiple-user monitoring of human respiration (Fig. 1b). Complex gestures and sign languages, and remote defusing bomb (Fig. 1b) are also demonstrated with a human-machine interface. All these functions have been achieved using a sensor-integrated glove to remotely control an external manipulator. This work exhibits great potentials of the flexible sensor for real time/long-range medical diagnosis and remote assistance to perform dangerous tasks in military and industry fields.

## Results and discussion

### Preparation of strain sensor

Figure 1a schematically illustrates the proposed two-step method with the interface engineering strategy based on surface wrinkles generated on Ecoflex, and the fabrication procedures of graphene/Ecoflex composite strain sensors. More details about the fabrication parameters and procedures can be seen in the Supplementary Fig. 1.

The first step of interface engineering method is to use an ethanol assisted solidification method to generate micron-sized (features of 5∼10 μm) wrinkles on Ecoflex after the volatilization of ethanol as shown in Fig. 1a. To do this, Ecoflex 00–30 A and B components with each weight of 10 g were mixed with 2.5 ml ethanol and cured at 30 °C for about 3 h to generate a solid Ecoflex elastomer.

The second step of the interface engineering method is to use a soaking method by putting the Ecoflex into a petroleum ether to form nano-sized wrinkles. To do this, the solid Ecoflex elastomer was immersed in a petroleum ether solution for 3 h, and thus the swollen Ecoflex elastomer was formed with its dimension increased to ∼1.7 times, and nanoscale wrinkle features of ∼250 nm were formed on the surface after shrinkage.

To verify the superior properties of our proposed graphene conductive materials and compare the matching between the conductive carbon materials and substrate, we have used the CNT and graphene nanoflakes (both are high performance nano-carbon based materials) as the conductive materials to fabricate strain sensors for comparisons. All the other preparation processes are kept the same for all the prepared samples. The experimental results are shown in the Supplementary Fig. 2, which demonstrates that applying graphene shows a better performance compared with CNTs.

Graphene nanoflakes with a weight of 0.06 g were added into a solvent of N-methyl-pyrrolidone (NMP, volume of 3 ml) and deionized water with a mixed volume ratio of 1:4. The mixed solution was then ultrasonically agitated for 3 h. The function of NMP was to dissolve the graphene nanoflakes uniformly in the solution, and the role of water was to reduce the solubility of graphene in the NMP, thus effectively transferring the graphene nanoflakes from NMP onto the Ecoflex (See Supplementary Note 3). To investigate the influences of graphene concentration and ratio of NMP/water on the performance of graphene/Ecoflex devices, we varied the graphene concentrations from 1, 2, 3, 4, to 5 mg/ml, and the ratios of NMP/water from 1:0.5, 1:1, 1:2, 1:3, 1:4, to 1:5. (See Supplementary Fig. 3).

The prepared wrinkled Ecoflex was then immersed into the mixed graphene: NMP: water dispersion solution, which was stirred for 3 h using a magnetic stirrer at a stirring rate of 800 rpm. This promoted the graphene flakes to be attached onto the surface of Ecoflex substrate. The graphene/wrinkled Ecoflex was then ultrasonically cleaned using the deionized water for 30 min, and then dried at 85 °C (in a vacuum oven) for 3 h to obtain the strain sensor. To investigate the effectiveness of interface engineering strategy and changes of Ecoflex on the performance of the graphene/Ecoflex sensors, we fabricated four types of Ecoflex samples, which are named as Ecoflex A, Ecoflex B, Ecoflex C and Ecoflex D, and their process conditions are listed in Table 1.

**Table 1:** The process conditions for four types of Ecoflex composite sensors.

Samples	First-step: With Ethanol assisted solidification	Second-step: Immersing into petroleum ether
Ecoflex A	No	No
Ecoflex B	Yes	No
Ecoflex C	No	Yes
Ecoflex D	Yes	Yes

### Properties of graphene/Ecoflex strain sensor

Figure 1c presents images of graphene/Ecoflex strain sensors with the stretched strains of 0%, 300 and 650%, respectively, demonstrating its good stretchability. Figure 1d illustrates initial resistance of four types of graphene/Ecoflex composite sensors fabricated in graphene: NMP: water solutions with an NMP/water ratio of 1:4 and a graphene concentration of 4 mg/ml. Results show that the resistance values of both graphene/Ecoflex B (applied with only the first-step surface treatment design) and graphene/Ecoflex C (applied with only the second-step interface modification engineering design) are smaller than that of graphene/Ecoflex A, because the substrates of Ecoflex B/C have been either modified with ethanol, or soaked inside the petroleum ether. More importantly, the graphene/Ecoflex D (applied with both two-step interface modification engineering designs) shows the smallest resistance value. Figure 1e shows the calculated relative resistance changes (∆*R*/*R*_0_) of these four types of graphene/Ecoflex sensors. The GF values of the strain sensors were calculated using (∆*R*/*R*_0_)/strain ε. The sample of graphene/Ecoflex D has the largest values of GF, and it has three different regions within the whole sensing range (with the estimated average GF values of 21.0, 191.6, and 1078.1, in the strain range of 0–200%, 200–450% and 450–650%, respectively.). The deformation tests at small strains (Supplementary Fig. 4) further prove that our sensor can detect 1% micro strain due to its high GF.

### Morphology Characterization and Sensing Mechanism

Supplementary Fig. 5 presents the Raman spectrum (WITec alpha300 R) and transmission electron microscope (TEM, FEI Tecnai G2 F20) image of the graphene used in this work, which verify that the graphene has a multilayer structure. The calculated defect density *n*_D_ is shown in Supplementary Note 6, indicating a defect density value of about 1.5867 × 10^11^ (cm^−2^). An atomic force microscope (AFM, Bruker Dimension ICON) was used to scan one graphene flake, and the obtained AFM image is shown in Supplementary Fig. 6. From the cross-section line along the height, the graphene flake has a thickness of ~1.5 nm and a lateral size of ~1 μm.

**Fig. 2:**
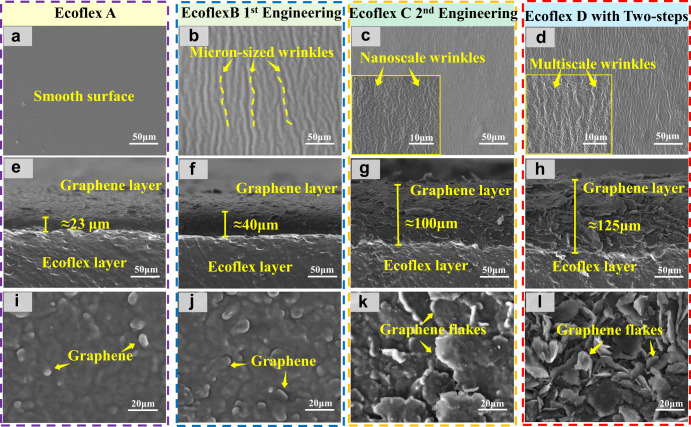
The SEM characterization of different types of graphene/Ecoflex. SEM images of surfaces of **a** EcoflexA, **b** EcoflexB, **c** EcoflexC, **d** EcoflexD; SEM images of cross-sectional morphologies of **e** graphene/EcoflexA, **f** graphene/EcoflexB, **g** graphene/EcoflexC, **h** graphene/EcoflexD; SEM images of surfaces for **i** graphene/EcoflexA, **j** graphene/EcoflexB, **k** graphene/EcoflexC, **l** graphene/EcoflexD.

To investigate the mechanisms of interface engineering strategy, surface and cross-section morphologies of different types of graphene/Ecoflex composites were characterized using a scanning electron microscope (SEM, TESCAN Company). Figure 2a presents the SEM images of surface morphologies of the untreated substrate (Ecoflex A). After applied with the first-step surface treatment, the surface of Ecoflex B shows many large micron-sized wrinkles as shown in Fig. 2b and Supplementary Fig. 7. These microscale wrinkles are mainly formed when the ethanol is rapidly volatilized, thus causing an uneven surface morphology^37^. This results in an increase in the surface area of Ecoflex and enhances the interfacial bonding between Ecoflex and graphene, promoting more graphene to be attached onto the surface of the Ecoflex^38^. The enhancement effect is strongly related to the discretization of the interfacial contact, termed as ‘contact splitting’, which is widely reported^39–42^. Cross-section SEM morphologies of different graphene/Ecoflex composites can prove this assumption, as the formed conductive sensitive layer of graphene on Ecoflex B (Fig. 2f) is much thicker than that of Ecoflex A (Fig. 2e). These microstructure changes will substantially decrease the resistance and increase the conductivity of the composite.

When only the second-step interface engineering method is applied, the surface of Ecoflex C shows many nanoscale wrinkles on the Ecoflex surface (Fig. 2c). This is because when the Ecoflex is soaked with petroleum ether, the petroleum ether molecules are diffused into polymers, causing the swelling effects. This results in the generation of nanoscale wrinkles after shrinkage^43^, and significantly increases the thickness of the conductive and sensitive layer on the surface of the Ecoflex (Fig. 2g), if compared with that of Ecoflex A (Fig. 2e).

When the Ecoflex was surface-engineered using the combined two-step strategy, a hierarchically multiscale wrinkles are formed on the Ecoflex surface as shown in Fig. 2d. This will greatly increase the specific surface areas of Ecoflex, and enhance the adsorption of more graphene, thus leading to the formation of a thicker conductive sensitive layer of graphene on the Ecoflex D (Fig. 2h) and the smaller value of resistance and larger conductivity of graphene/Ecoflex composite. Supplementary Fig. 8 compares the differences of graphene conductive layer thickness for different samples at different magnifications. Figure 2i∼l show the surface morphologies of different graphene/Ecoflex composites, with or without applying interface engineering strategy. Results show that the graphene adsorbed onto the Ecoflex composite treated with our two-step strategy appear in the form of self-overlapping graphene flakes, thus providing a good conductivity and sensitivity.

There are many studies to explore the sensing mechanisms of graphene-based strain sensors^44^. For example, Hempel et al. reported that the conductivity among the neighboring graphene flakes is influenced by contact resistance and overlapping areas. After the strain is applied, the neighboring flakes are gradually separated, thus breaking the percolation pathways and causing a singularity in the resistance-strain behavior^45^. Hassan et al. reported that the graphene strain sensor’s electrical resistance was changed because of: (a) the decreased overlapping areas between the graphene flakes under a small strain; and (b) the propagating cracks in the conductive network under a large strain^46^.

**Fig. 3:**
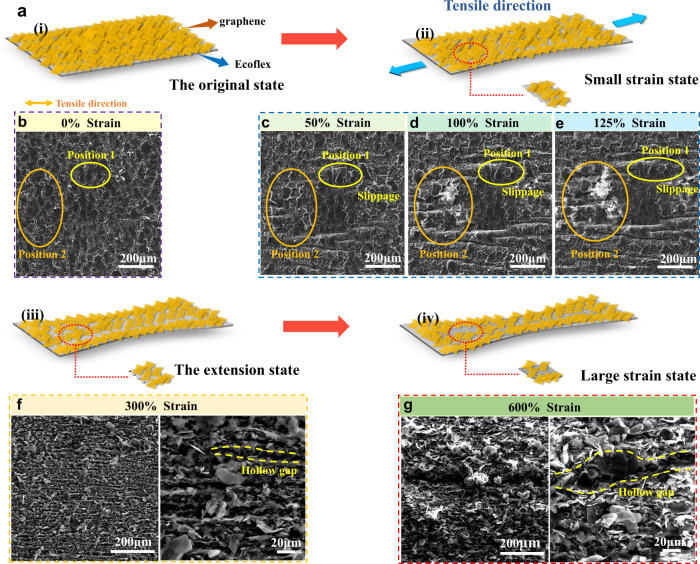
The resistance sensing mechanism of graphene/Ecoflex strain sensor. **a** Schematic illustrations of resistance sensing mechanism of graphene/Ecoflex strain sensor under a stretching process. Top-view SEM images of graphene/Ecoflex strain sensor with various strain: **b** 0%, **c** 50%, **d** 100% and **e** 125% **f** 300% and **g** 600%.

To explain the sensing mechanism for our case, we illustrate the sensing mechanism based on the resistance changes under stretching processes for the graphene/Ecoflex composite strain sensor, as shown in Fig. 3a. The strain sensing process can be classified into four steps. (i) Dense and self-overlapping graphene flakes are formed in their original state. (ii) With the increase of strain, there are some minor changes of self-overlapping graphene flakes and appearance of some slippages and displacement movements. Some neighboring flakes are gradually separated, thus losing their close contact. The breakage of percolation pathways occurs under such a strain. (iii) Under a relatively large strain, the phenomena include extension and separation of self-overlapping graphene flakes and the formation of gaps within the conductive graphene layer. (iv) Finally there are severe separations of the previously overlapped graphene flakes and formation of larger hollow gaps in the graphene layer under a large strain.

To further investigate the sensing mechanism, we conducted in-situ tensile tests within the SEM chamber to observe the morphology of the graphene/Ecoflex under various strains. We firstly increased the applied strains from 0%∼125%, and the strain was limited to 133% due to the limitation of the equipment. We also performed the tests with fixed tensile strains of 300 and 600%. The obtained results are shown in Fig. 3b∼g. In the unstretched state of the sensor, many graphene flakes are self-overlapped and densely packed on the surface of the film (Fig. 3b), and thus a conductive network is formed to provide the composite with a low resistance.

When the sensor is under a low strain (for example, below 125%), the overlapping areas among flakes are decreased as shown in Fig. 3c–e. Some of the graphene flakes have shown slippages and displacement movements. Many neighboring flakes are gradually separated. The breakage of percolation pathways occurs, leading to an increase of the resistance. In this stage, the graphene flakes are still overlapped and mainly horizontally stacked from the bottom layer to the upper layer, and there are still good contact among neighboring flakes. Therefore, the percolation pathways of the graphene flakes are effective. The variation of resistance is relatively small, and the GF value of the sensor is low.

With the application of a larger tensile strain such as 300% (Fig. 3f), the graphene flakes become significantly separated, and the stacked patterns of some graphene flakes are changed from horizontal to vertical or disordered patterns. Therefore, the original overlapped graphene flakes are dramatically separated, and the overlapping areas are continuously decreased under the large strain, leading to the increased strain sensitivity. During this stage, some ridges on the surface of strain sensor are emerged and these ridges cause hollow gaps in the conductive graphene layer. These two reasons will result in a significant reduction of the conductive channels and a significant increase in strain sensitivity.

When the strain is increased to 600% (Fig. 3g), the graphene flakes become severely separated and disordered, and the overlapping areas among the flakes are decreased sharply. In addition, the film becomes much thinner, and the gaps within the graphene conductive layer are significantly enlarged. These lead to more significant increase of the resistance and a remarkably large GF value.

### Electromechanical Performance of Graphene/Ecoflex strain Sensor

Supplementary Fig. 3 presents the optimized parameters of graphene concentrations and NMP/water ratios for the graphene/Ecoflex composite sensors. Results show that the optimal graphene concentration is ~4 mg/ml and the optimal NMP/water ratio is 1/4. These parameters were chosen to prepare the sensors.

**Fig. 4:**
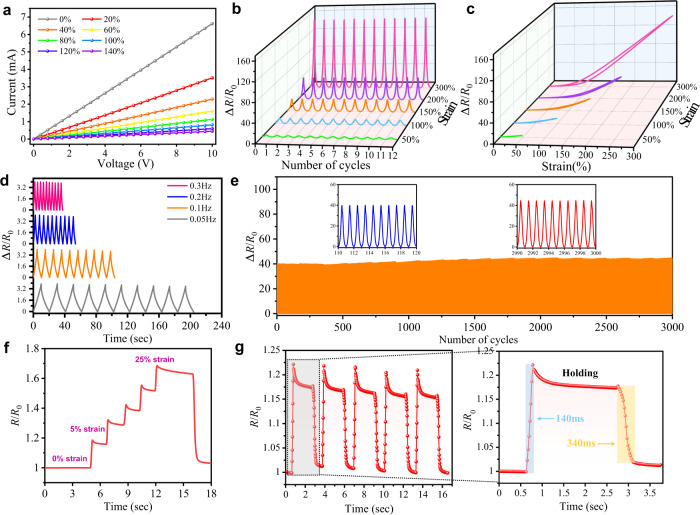
Electromechanical characterization of the graphene/Ecoflex strain sensor. **a** *I–V* curves of the strain sensor under various strains from 0 to 140%. **b** The change in relative resistance of the sensor during 12 cycles of loading−unloading under different strains (50 %∼ 300%). **c** Hysteresis curve of the sensor under different strains. **d** Relative resistance response of the strain sensor at different frequencies under a strain of 50%. **e** The cycling durability curve of the strain sensor under 200% strain. **f** The real-time *R*/*R*_0_ curve of the strain sensor when loaded with different strains. **g** The real-time *R*/*R*_0_ curve of the strain sensor under repeated loading 5% strains and the corresponding response and relaxation time.

We have measured the results of current versus voltage (*I–V*) for the strain sensor under varied strains. As shown in Fig. 4a, by stretching the sensor from its initial length to a strain of 140%, all the *I–V* curves reveal linear relationships, demonstrating that the resistance of the sensor remains quite stable with the change of voltage under different strains from 0 to 140%. The slopes of these *I–V* curves are decreased with the increased external strain, due to the increased resistance values with the increased strain.

Figure 4b presents variations of the relative resistances for the graphene/Ecoflex strain sensor imposed with different tensile strains of 50, 100, 150, 200, and 300%. The sensors exhibit a good resistance response and display a good strain stability over a wide strain range (50∼300%). Figure 4c shows the corresponding hysteresis curves of the sensor, and the stretching and releasing curves are overlapped when the strain is less than 200%, highlighting negligible hysteresis in the resistance changes under different mechanical loading conditions. With a larger strain ε of 300%, the hysteresis is relatively large. However, the resistance could be almost recovered to its original state after stretching, illustrating the good stretchability of the proposed sensor. Formation of the hysteresis of the resistance curves is mainly due to the different time scales which are associated with the breakdown and reformation of contacts between graphene flakes within the network, as well as the inherent hysteresis associated with elastomeric loading^47–49^. Fig. 4d and Supplementary Fig. 9 show the frequency responses and output signals of the strain sensor with the frequencies ranging from 0.05 to 0.3 and to 0.5 Hz at a strain of 50%. Results clearly show that the sensor shows good dynamic characteristics within the frequency range from 0.05 to 0.5 Hz. With the increase of frequency, the peak variations of the relative resistances are almost the same. This is mainly due to the good adhesion and dynamically interfacial bonding of hierarchical wrinkling patterns between the graphene and Ecoflex.

To evaluate the sustainability and long-term stability, we applied the same device with a maximum strain of ε = 200% for 3000 cycles of repeated stretching/releasing processes at a stretching–releasing sweeping speed of 100 mm min^−1^. Figure 4e exhibits that the electrical response is reproducible throughout the overall fatigue test with slight fluctuation, demonstrating its good repeatability, stability, and durability. Under a 300% loading-uploading strain, the proposed sensor can still retain a signal amplitude with repetitive strain loading up to 1500 cycles (Supplementary Fig. 10).

The response time of the sensor was also obtained with the loading strain changed with a sequence of 0–5–10–15–20–25–0%, and also with a fast repetitive response and release process at a fixed 5% strain. The obtained results are illustrated in Fig. 4f & g. The response time of our developed strain sensor is as short as 140 ms (Fig. 4g). The release time is slightly larger (~340 ms) due to its viscoelasticity and hysteresis. The sharp overshoots are observed as shown in Fig. 4g, which is mainly due to the tensile stress-relaxation under the applied strain, caused by the viscoelasticity of the Ecoflex composite^50–52^.

Temperature effect on the resistance changes was also investigated, and the obtained results are shown in Supplementary Fig. 11. The obtained experimental data clearly show that temperature can slightly cause the changes of the resistance. It does not have an apparent influence on the conductivity of the sensor, compared with the effect of strain. We think the possible methods to minimize or eliminate the influence of temperature could be: (1) adopting a reference device, or (2) using a thermal isolation package.

We have compared the performance of various resistance-type stretchable strain sensors reported in literature with that of our work, and the results are summarized in Table 2. Clearly our fabricated device has a combined good performance with a high stretchability, large sensitivity, and fast response time at the same time. Compared with previously reported methods of flexible strain sensors, our methodology has its pros and cons. The proposed process mainly involves a solution treatment method, which does not involve any expensive equipment and/or complicated preparation processes. Besides, by precisely controlling the solution concentration and treatment durations, high performance samples can be easily obtained. Although the sensitivity of our sensor is slightly less than those based on microcrack mechanism^28,30^, our sensor’s detection range is much wider, which indicates that our device can be used for a wide-range applications of both large and small strain scenario. Of course, our sensor and its process still have some limitations. Firstly, compared with those based on a single step treatment, our two-step treatment method could increase the time and cost of preparation process. In addition, our method is not IC compatible. Finally, the strain sensor shows overshoots during its usage, which is mainly due to the tensile stress-relaxation behavior under the applied strains.

**Table 2:** Comparisons of Device Performance of Resistance-Type Strain Sensors.

Sensor type	Gauge Factor	Stretchability (%)	Response Time (ms)	Cyclic Stability	Ref.
CB/Ecoflex	67.7 (300–500% strain)	500	120	5000 cycles (0–150%).	18
				1390 cycles (0–200%)	
CNT/PDMS	87 (0–40% strain)	100	65	1500 cycles (0–20%)	19
	6 (40–100% strain)				
MWCNT/TPU fiber	22.2 (<160%)	320	200	9700 cycles (0–100%)	21
	97.1 (160–320%)				
PDA/CB/NBR	346 (160–180% strain)	180	/	1000 cycles (0–20%)	24
rGO/PDMS	88443(300–350%)	350	145	5000 cycles (0–50%)	25
CNT/PDA/elastic bands	129 (780–920%)	920	220	10,000 cycles (0–100%)	26
Fish-scale rGO/elastic tape	16.2 (<60% strain)	82	/	>5000 cycles (0–10%)	27
	150 (>60% strain)				
Microcracks Graphite/Ecoflex	522.6 (short microcracks)	≥50 (short microcracks)	/	unmentioned	28
	11344 (long microcracks)	≤50 (long microcracks)			
Microprism-array AgNW/elastomer	81 (>130% strain)	150	/	10,000 cycles (0–150%)	29
Acid-interface engineering of CNT/Ecoflex	35.2 (0–80% strain), 129.1 (80–120% strain), 1665.9 (>120% strain)	>100	/	10,000 cycles (0–80%)	30
Graphene/wrinkled Ecoflex	1078.1 (450–650% strain)	650	140	3000 cycles (0–200%)	This work

*TPU* thermoplastic polyurethane, *PDA* polydopamine, *NBR* nitrile butadiene rubber.

### Human motion detection and cloud platform monitoring

**Fig. 5:**
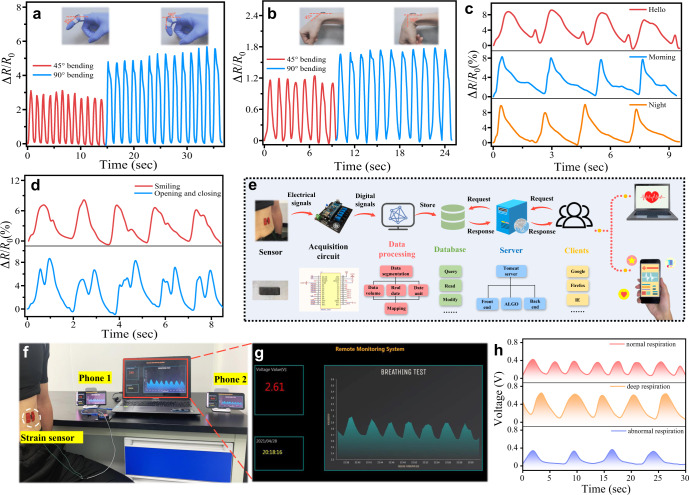
Wide-range physiological signals detection and respiration monitoring using a cloud platform. Relative resistance changes with **a** index finger bending, **b** wrist bending, **c** phonating (‘Hello’, ‘Morning’ and ‘Night’), **d** subtle facial expressions movements (smiling and opening and closing mouth). **e** Schematic illustration of big data cloud monitoring platform. **f** Photograph showing the strain sensor placed on the abdomen for measuring the respiratory signal. **g** Big data platform display interface **h** Three different respiration signals of a volunteer on the cloud data platform.

The sensor was attached to different parts of one volunteer’s body, e.g., knuckle (Fig. 5a), wrist (Fig. 5b) and elbow (Supplementary Fig. 12a), in order to monitor body motions. Figure 5a shows the resistance changes from the movement of the index finger. When the finger was bent up, the resistances of the sensor were increased. Further bending up to 90° would lead to a significant increase in the resistance. The results in Fig. 5a demonstrate good stability and repeatability of the data. We have further done similar measurements with the sensors attached on the wrist (Fig. 5b), and the sensor shows good repeatable results with a continuous bending.

The strain sensor was further attached to the throat of a volunteer to monitor the sound (Fig. 5c). It can detect various sound words, such as ‘Hello’, ‘Morning’ and ‘Night’, and the response curves exhibit characteristic patterns with a good repeatability. The sensor can also distinguish the minor differences in the sound/pronunciation such as ‘Quite’ ‘Quiet’, as shown in Supplementary Fig. 13. Our studies show that the developed strain sensor has a great potential in aided speech rehabilitation training.

This strain sensor can also capture the volunteer’s breathing and swallowing motions (Supplementary Fig. 12b). When the strain sensor was attached near the cheek, it can capture subtle facial expressions and movements (Fig. 5d), including smiling, opening and closing of mouth. For monitoring tiny physiological signals, the wearable strain sensor was attached onto a volunteer’s wrist and the wrist pulses under both relax and exercise conditions were measured. The obtained results are shown in Supplementary Fig. 12c, which reveal the significant differences in different conditions.

To demonstrate the WIoT functionality and real-time, remote and multiple-user monitoring, we designed a data cloud platform and integrated our sensor with the data cloud platform to remotely monitor human breathing state in real time for multiple users. The schematic illustration of big data cloud monitoring platform is shown in Fig. 5e. The breathing status of a person can reveal symptoms of several serious diseases (e.g., novel coronavirus pneumonia such as COVID-19 and central sleep apnea). Previous studies of real-time monitoring were mostly based on Wi-Fi or Bluetooth, whereas our real-time remote monitoring is based on big data cloud platform without any distance limitation and can also be easily accessed by multiple users. Figure 5f, g and the Supplementary Video 1 exhibit the experimental test results, demonstrating that the cloud data platform based on our proposed sensor can easily distinguish patterns of different respiratory states such as normal respiration, deep respiration and abnormal respiration over a long distance (Fig. 5h). Therefore, our flexible strain sensor can obtain stable and accurate respiratory signals, and provide critical physiological data of cardiovascular systems for the monitoring and evaluation of the associated diseases, over a long distance.

### Gesture recognition

**Fig. 6:**
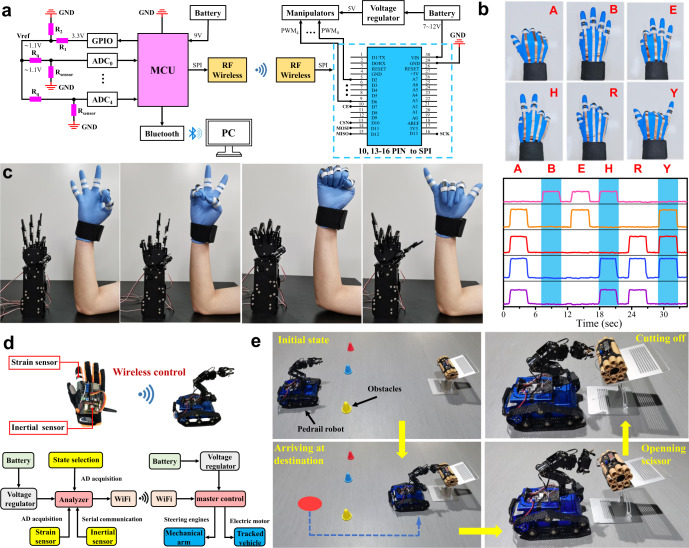
Applications of graphene/Ecoflex strain sensor in human-machine interface. **a** Circuit scheme showing the signal transduction, processing, and wireless transmission by using graphene/Ecoflex strain sensors to realize gesture recognition and gesture control. **b** Photographs of a ‘data glove’ mounted with the assembled graphene/Ecoflex strain sensors to perform a series of gestures and the relative resistance changes of each strain sensor when expressing the letters ‘A’, ‘B’, ‘E’, ‘H’ ‘R’ and ‘Y’. **c** Photographs of gesture control. **d** Schematic diagram of smart glove and pedrail robot and control process. **e** Photographs of the motion path of the pedrail robot and the thread trimming process.

To further demonstrate the potential of our developed flexible graphene/Ecoflex strain sensors for human-machine interface application, a sensor-integrated smart glove was fabricated, and the corresponding circuit was designed and fabricated. This smart glove can recognize hand gestures and real-time control the external manipulator, and the results are shown in Fig. 6a. The system included four parts: i.e., the data acquisition unit, data processing unit, data transmission unit and control unit. The data acquisition unit was consisted of a voltage divider circuit and five analog-to-digital convertor (ADC) channels, which converted the resistance signals into voltage signals and then into the digital voltage data. Five graphene/Ecoflex strain sensors were attached onto each finger of the glove and wired to the acquisition circuit which was attached on the glove. When the finger was bent or straightened, the resistance of the corresponding strain sensor was acquired and recognized. The data processing unit was consisted of a high-performance MCU (microcontroller unit), equipped with an ATmega328P chip. The MCU was powered by a battery and provided an output of 3.3 V port-voltage. The data transmission unit was consisted of a Bluetooth module and an RF module, and the response data were transmitted via a Bluetooth module and recorded by a PC wirelessly. The control unit included a wireless radio frequency module, a controller module (Arduino nano) and a manipulator with several motors which mimicked a series of human movements.

We demonstrated the hand gesture recognition using the graphene/Ecoflex strain sensor. Six hand gestures which come from Sign Languages have been applied to represent the characters ‘A’, ‘B’, ‘E’ ‘H’ ‘R’ and ‘Y’, respectively, as shown in Fig. 6b. These six Sign Language characters can be easily recognized and presented according to the response curves of the five graphene/Ecoflex strain sensors. This demonstrates its great potential applications in sign language recognition, beneficial for those hearing-impaired people.

The graphene/Ecoflex strain sensors were further integrated into a wireless-controlled, human–robot interactive systems. Five strain sensors were mounted on a thin glove and connected with a single-board microcontroller, as shown in Fig. 6c and the Supplementary Video 2. We have successfully demonstrated the dynamic controlling and multichannel sensing functions, proving the potential applications into the human–robot interactive systems.

### Remote defusing bomb

Finally, we designed a remote defusing bomb control system, where the movements of the pedrail robot were wirelessly and controlled by a tri-axis inertial sensor. Our strain sensor was attached to a finger of glove to execute the command of defusing bomb using a scissor, as illustrated in Fig. 6d. Notably, the pedrail robot was driven to avoid obstacles and reach a target location by hand movements, while the power wire of the mock bomb was cut off using the scissor by simply bending the finger (Fig. 6e and Supplementary Video 3), displaying the great application prospects in intelligent vehicles designed for remote assistance to perform extreme and dangerous tasks in the military field, or for vulnerable groups such as the disabled and elder people.

In this study, we proposed a methodology by modulating multiscale and hierarchical wrinkles on flexible substrate to be integrated into strain sensors based on graphene/Ecoflex composites. The optimized design and manufacturing endow the sensor with an ultra-high sensitivity with a GF of 1078.1, a large stretchability up to 650%, a fast response with a time of ~140 ms, and superior cycling durability. The sensor can detect wide-range physiological signals including vigorous body motions, pulse monitoring and speech recognition. We applied the proposed sensors for remote monitoring respiration for multi-users in real-time using a cloud platform, showing its great potentials in wearable health internet of things. Complex gestures and sign languages were readily detected using our sensor. We further demonstrated human-machine interface by using a sensor-based glove to remotely control an external manipulator and realized function of remotely defusing a bomb. Our results shed light on the application of real time and long-range medical diagnosis and remote assistance to perform dangerous tasks in the military and industry fields.

## Methods

### Characterizations

Material properties of the graphene used in this work were characterized using a Raman spectroscope (WITec alpha300 R) and a transmission electron microscope (TEM, FEI Tecnai G2 F20). The average sheet size of graphene flake was measured using an atomic force microscope (AFM, Bruker Dimension ICON). Cross-section morphology and surface topography of graphene/Ecoflex samples were characterized with a scanning electron microscope (SEM, TESCAN Company). Tensile stress-strain measurement of the graphene/Ecoflex composites were conducted at room temperature using a tensile testing machine (ZQ-990A), at a loading rate of 5 mm s^−1^ and a clamp distance of 1 cm. Electrical signals of the sensors were measured using a Keithley 2611B source meter.

### Wide-range physiological signals detection

The proposed graphene/Ecoflex strain sensors were attached to various places (e.g. wrist, knuckle, elbow, abdomen, throat and face) of a volunteer using medical tapes. Movements of joints and mechanical signals of skin deformation were detected using the graphene/Ecoflex strain sensor with Keithley 2611B source meter.

### Gesture recognition and real-time control of external manipulators

Five flexible graphene/Ecoflex sensors were mounted onto each finger of a glove and wired to the acquisition circuit. The response data were collected through five ADC channels and wirelessly transmitted through a HC-05 Bluetooth module to the computer. The data were processed by characteristic engineering to monitor the hand gestures. For the real-time control of external manipulator systems, the response data were collected from each sensor using a designed chip (which reflect the states of the five fingers) and then transmitted wirelessly to a computer. The respective motors of the robot arm were wirelessly activated based on a thresholding algorithm, using the sensor integrated glove.

### Cloud-platform remote and multiple-users monitoring

To demonstrate the WIoT functionality and real-time, remote and multiple-user monitoring, we designed a data cloud platform to achieve real-time monitoring of human respiration. Compared with using Bluetooth or WiFi based real-time monitoring (which normally has a distance limitation), the cloud-platform monitoring in this study can truly realize remote monitoring, which can be realized in the world wide web without any distance restrictions, as long as the users have an internet connected. Another advantage is that the data can be accessed by multiple users within world-wide. In this work, we attached our fabricated sensor on the volunteer’s abdomen to remotely monitor respiration. The obtained data were analyzed using voltage divider circuit, A/D conversion and signal processing modules, then they were sent to the database to be stored and further transmitted to a cloud platform which was built by us. The data were displayed in real-time on the cloud platform interface and the multi-users could view the data in real-time through remotely access on Uniform Resource Locator (URL) links.

### Remote defusing bomb

For demonstration of remote defusing bomb, our flexible strain sensor was combined with a tri-axis inertial sensor in order to detect finger bending motions and three-dimensional motions of a person’s arm. The data from the body-worn and tri-axis inertial sensor were used to wirelessly control the movements of the pedrail robot (forward moving, back moving, left turning, and right turning) and the robotic arm’s movements, and the selection for controlling pedrail robot or robotic arm was operated by a channel switch. The flexible graphene/Ecoflex strain sensor was mounted onto the index finger of the above glove, and the response data from flexible sensor can be wirelessly transmitted to a motor in the robot arm, thus realizing the function of defusing the bomb.

## Supplementary information

Supporting information

Supplementary Video 1 Respiratory monitoring

Supplementary Video 2 Manipulator control

Supplementary Video 3 Remote bomb removal

## Data Availability

The data that support the findings of this study are available from the corresponding author upon reasonable request.
