# Supplementary material for: Multiscale and hierarchical wrinkle enhanced graphene/Ecoflex sensors integrated with human-machine interfaces and cloud-platform
Source: Npj Flex Electron. 2022 Jul 5;6(1):55. doi: 10.1038/s41528-022-00189-1 (PMC9255543; doi:10.1038/s41528-022-00189-1)
Supplement: Supplementary file 1 — Supporting information [file 41528_2022_189_MOESM1_ESM.pdf]

Supporting Information

Multiscale and Hierarchical Wrinkle Enhanced Graphene/Ecoflex Sensors Integrated with Human-Machine Interfaces and Cloud-Platform

*Jian Zhou[1], Xinxin Long[1], Jian Huang[1], Caixuan Jiang[1], Fengling Zhuo[1], Chen Guo[1], Honglang Li[2], YongQing Fu[3], Huigao Duan[1,4] **

1. College of Mechanical and Vehicle Engineering, Hunan University, Changsha 410082, China

2. CAS Center for Excellence in Nanoscience, National Center for Nanoscience and Technology, Beijing 100190, China

3. Faculty of Engineering and Environment, Northumbria University, Newcastle upon Tyne, NE1 8ST, United Kingdom

4. Greater Bay Area Institute for Innovation, Hunan University, Guangzhou 511300, Guangdong Province, China

Corresponding Emails: duanhg@hnu.edu.cn;

Supplementary Note 1. Fabrication process of graphene/Ecoflex strain sensor

Ecoflex 00-30 components of A and B with each weight of 10 g (purchased from the US Smooth-on Co., Ltd.) were mixed with 2.5 ml ethanol and then stirred for 5-10 minutes. The bubbles generated during the stirring process were removed using a vacuum pump (FY-1H-N, Zhejiang Feiyue Electrical Co., Ltd.). The prepared liquid Ecoflex was then transferred to the grooves formed on the mold printed using a 3D printer (Shenzhen Chuangxiang 3D Technology Co., Ltd.) and cured at 30 °C for 3 hours to form a solid Ecoflex elastomer. The shape of the mold was rectangular, with its length of 30 mm, width of 10 mm and height of 1.5 mm.

The fabricated solid Ecoflex was immersed in a petroleum ether solution for 3 hours, and the dimension of Ecoflex was increased to ~1.7 times, thus a swollen Ecoflex elastomer was formed.

Graphene with a weight of 0.06 g was added into a mixed solution of N-methyl-pyrrolidone (NMP, volume of 3 ml) and deionized water with a volume ratio of 1:4. The mixed solution was ultrasonically agitated for 3 hours in order to disperse the graphene nanosheet uniformly into the solution.

Finally, the prepared Ecoflex was immersed into the graphene:NMP:water dispersion solution, which was stirred at room temperature for 3 hours using a magnetic stirrer at a stirring rate of 800 rpm. This promotes the graphene attached onto the surface of Ecoflex elastomer. The prepared sample was ultrasonically cleaned with the deionized water for 30 min, and vacuum-dried in a vacuum oven at 85°C for 3 hours. Then, the graphene/Ecoflex strain sensor was obtained. (Supplementary Figure 1)

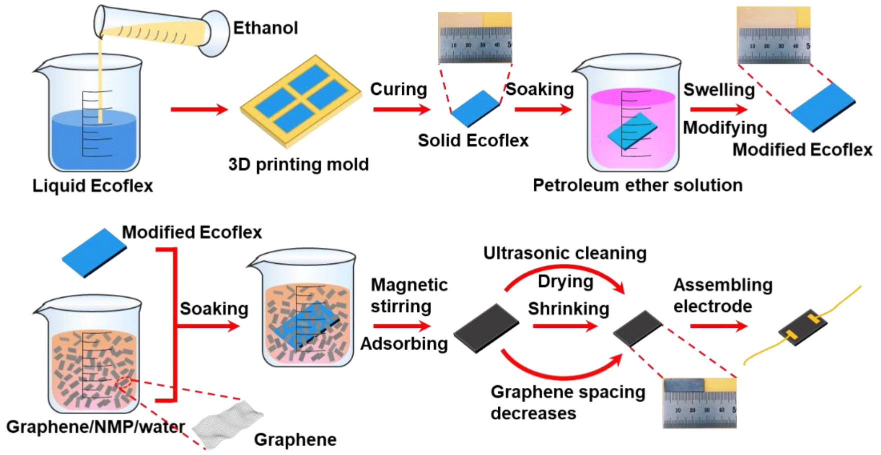

Supplementary Figure 1. The detailed fabrication procedures Schematic illustration of graphene/Ecoflex strain sensor.

Supplementary Note 2. A comparative study of graphene/Ecoflex Sensors and CNT/Ecoflex Sensors

The conductive properties of materials and a good matching between the conductive materials and substrate is very important. In order to investigate the effects of different conductive materials on the performance of sensor, we have used CNT and graphene (both are high performance nano-carbon based materials) as the conductive materials for strain sensors for comparisons. We kept the same experimental parameters for the other preparation processes. The experimental results are shown in Supplementary Figure 2. It is clear that our proposed wrinkle enhanced graphene/Ecoflex sensors show much better performance compared with that of wrinkles enhanced CNT/Ecoflex sensors. The GFs of CNT/Ecoflex Sensors strain sensors are only 5.7 (ε: 0-200%) and 16.3 (ε: 200-650%), which are much smaller than those of our graphene/Ecoflex sensors. This clearly demonstrates that the conductive materials and a good matching between the conductive materials and substrate are the key factors to achieve the high quality strain sensors.

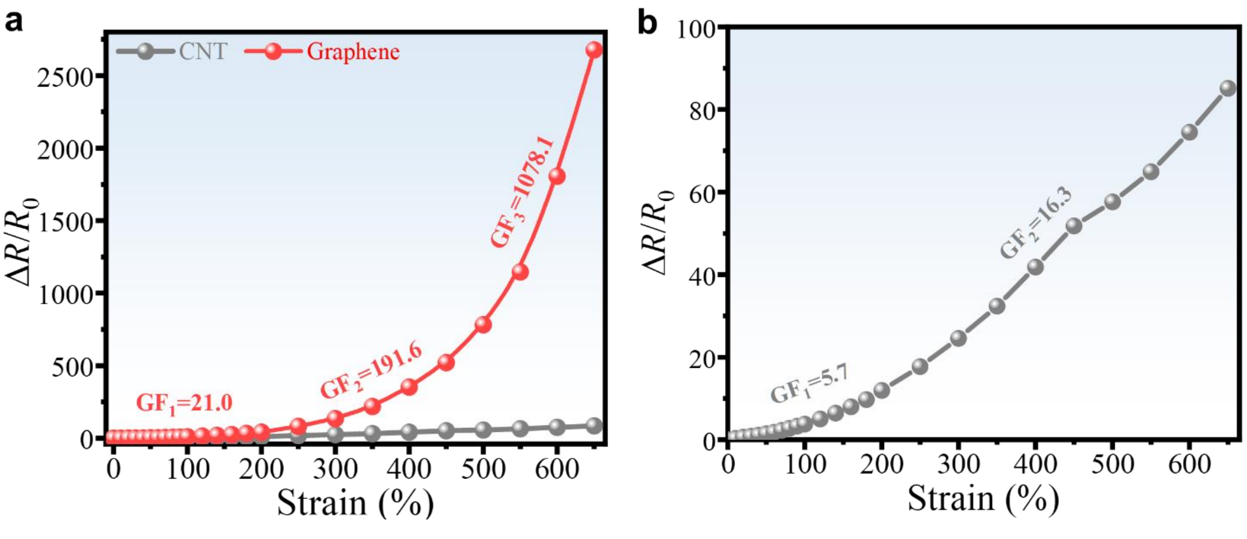

Supplementary Figure 2. a The relative resistance changes ($\Delta R/R_0$) versus strain of CNT/Ecoflex and the graphene/Ecoflex strain sensor; **b** Enlarged Drawing of CNT/Ecoflex strain sensor.

Supplementary Note 3. Hansen solubility parameters of various materials in this study and their effects

Generally, for two substances to be mixed/interacted effectively in a molecular scale, their Hansen solubility coefficient parameters must be close to each other. [1] The Hansen parameters

of graphene (δ_D= 18 MPa$^{1/2}$, δ_P= 9.3 MPa$^{1/2}$, δ_H= 7.7 MPa$^{1/2}$) are much closer to those of NMP (δ_D=18 MPa$^{1/2}$, δ_P=12.3 MPa$^{1/2}$, δ_H=7.2 MPa$^{1/2}$), if compared with those of Ecoflex (δ_D≈13 MPa$^{1/2}$, δ_P=0 MPa$^{1/2}$, δ_H=0 MPa$^{1/2}$). Therefore, without the water involved, it is more energetically favorable for the graphene to be attached with NMP rather than the Ecoflex. In that case, it is unlikely that the graphene is effectively attached onto the surface of the Ecoflex.[2]

To address this problem, we made it less energetically favorable for the graphene to remain dispersed uniformly in the NMP by adding water into the graphene-NMP dispersion. The Hansen parameters of water (δ_D=15.5 MPa$^{1/2}$, δ_P=16 MPa$^{1/2}$, δ_H=42 MPa$^{1/2}$) are very different from those of the NMP. By adding the water into NMP, the Hansen parameters of NMP: water mixture have been changed significantly. These values are significantly different compared to those of the graphene, making it more favorable for the graphene to be attached onto the surface of Ecoflex. [3] The Hansen solubility parameters of various materials used on this study are listed in the Supplementary Table 1.

Supplementary Table 1. Hansen solubility parameters of various materials used on this study.

	**δ_D (MPa$^{1/2}$)**	**δ_P (MPa$^{1/2}$)**	**δ_H (MPa$^{1/2}$)**
**Ecoflex**	≈13	0	0
**Graphene**	18	9.3	7.7
**NMP**	18	12.3	7.2
**Water**	15.5	16	42.3

Supplementary Note 4. Effects of graphene concentration, NMP/water ratio of graphene/NMP dispersion solution on performance of graphene/Ecoflex strain sensors

Supplementary Figure 3 shows the initial resistance values of the composites prepared under the conditions of different NMP: water ratios and different concentrations of graphene dispersion. From Supplementary Figure 3a, we can see that when the graphene concentrations are 1 mg/ml, 2 mg/ml, 3 mg/ml, 4 mg/ml and 5 mg/ml, and when the ratio of NMP/water is greater than 1:4, the initial resistance of the composites decreases with the increase of the proportion of water in the graphene dispersion, which shows that adding water can effectively

transfer graphene nanoflakes from NMP onto Ecoflex. When the ratio of water in the graphene dispersion is further increased to ¼ of NMP/water, the resistance of the composites shows the smallest value. With the further increase of the water contents, the resistance of the composite becomes increasing. The possible reason is that large content of water causes the decrease of the solubility of graphene in the dispersion, and the graphene becomes agglomerated, thus affecting the adsorption of graphene by the Ecoflex. Therefore, the optimum proportion of NMP/water to achieve the best conductivity of the sensor is ¼ in this study. Moreover, when the concentration of graphene is 4 mg/ml and the ratio of NMP/water is ¼, the resistance of the sensor shows the smallest value. In addition, the resistance of the composites is quite high, but its strain detection range is low. The reason is that there are not many conductive paths. When the composites are loaded with a large strain, the conductive paths are easily broken, so the detection range is small. From Supplementary Figure 3b~f, we can see that when the ratios of NMP/water in the graphene dispersion are 1:3, 1:4 and 1:5, and the strain detection range of the prepared composite can reach up to 650%, indicating that water in graphene dispersion plays an important role in improving the sensing performance of the prepared composite. In addition, the composites with the graphene concentration of 1-4 mg/ml and the ratio of NMP/water of 1:4 has shown much higher sensitivities than those of the other ratios of NMP/water but the same graphene concentration value ranges. When the graphene concentration is 4 mg/ml and the NMP/water ratio is 1:4, the sensitivity of the composite reaches its highest value. In summary, the optimal ratio of NMP/water in graphene dispersion is 1:4 and the optimal concentration of graphene is 4 mg/ml in this study.

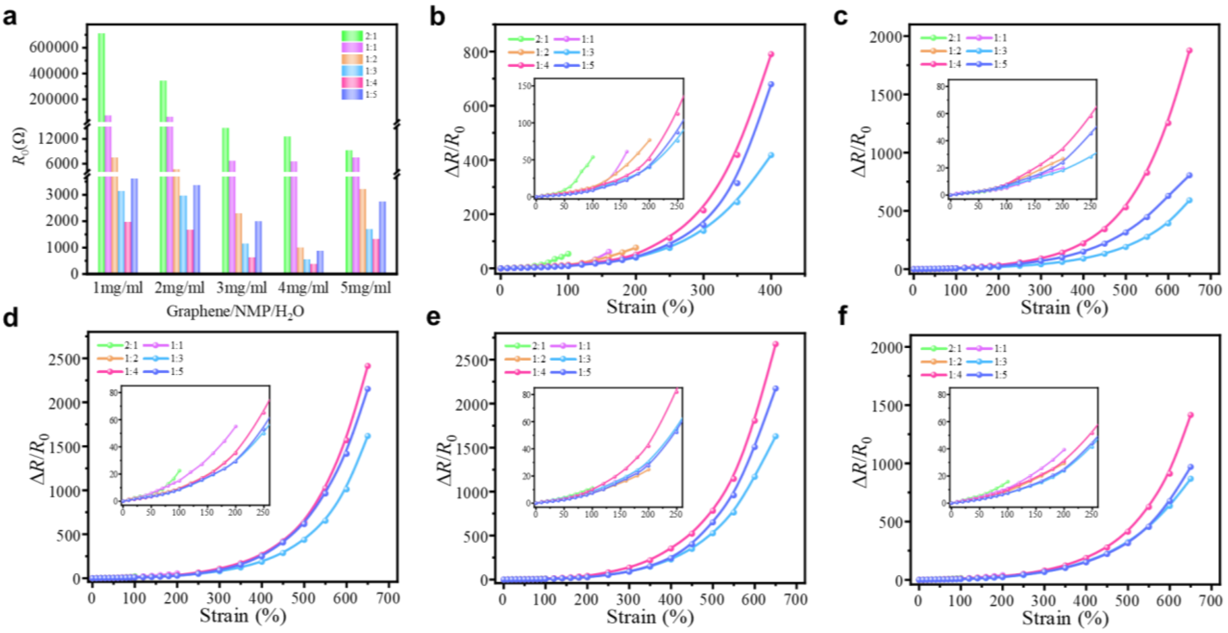

Supplementary Figure 3. Electrical properties of Graphene/Ecoflex composites prepared from different graphene dispersions. **a** Initial resistance of Graphene/Modified Ecoflex Composites; **b** When graphene concentration is 1 mg/ml； **c** graphene concentration is 2 mg/ml; **d** graphene concentration is 3 mg/ml； **e** graphene concentration is 4 mg/ml; and **f** graphene concentration is 5 mg/ml, Relative resistance changes of composites prepared by graphene dispersion with different NMP: water ratio.

Supplementary Note 5. The deformation tests of graphene/Ecoflex D

We further carried out the deformation tests using our sensor under the small strain. The experimental results are shown in Supplementary Figure 4. It is clear that our sensor can detect 1% micro strain and has shown a good linearity within a strain of 50%.

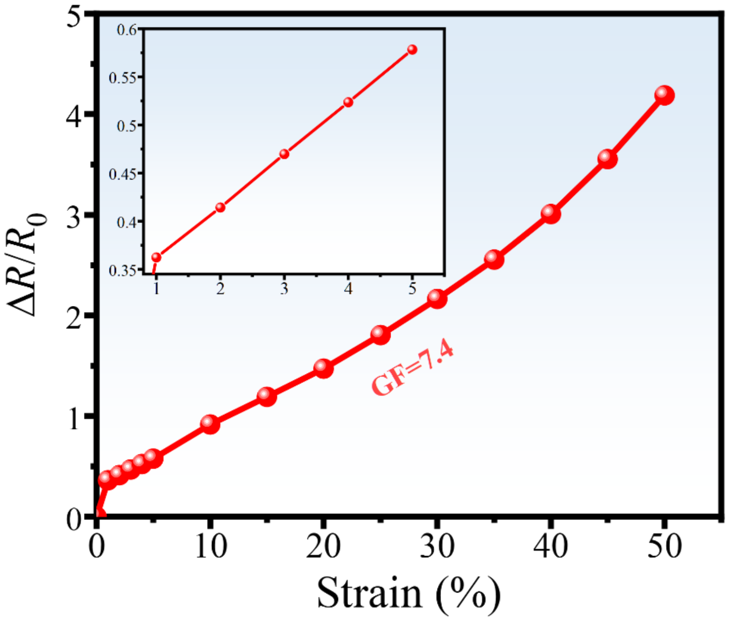

Supplementary Figure 4. The relative resistance changes ($\Delta R/R_0$) versus strain for graphene/Ecoflex

Supplementary Note 6. Detailed characterization results of graphene employed in this work

We have used Raman spectroscopy and transmission electron microscopy (TEM) to characterize the graphene employed in this work. Supplementary Figure 5a presents the results of Raman spectrum. It is clear that the graphene mainly exhibits two remarkable bands at around 1570 and 2694 cm^{-1}, which are corresponding to the G-band and 2D-band of graphitic carbon, respectively.[4] The relative intensity ratio (I_G/I_{2D}, where I_G and I_{2D} are the integral areas values of G-band and 2D-band,[5] respectively) is about 1.46, indicating that the graphene has a multilayer structure. The existence of a relatively weak D-band at 1341 cm^{-1} reveals the defects of the graphene. Supplementary Figure 5b presents the TEM results of graphene. It can be seen that the graphene has a multilayer structure, which is consistent with the Raman spectrum result.

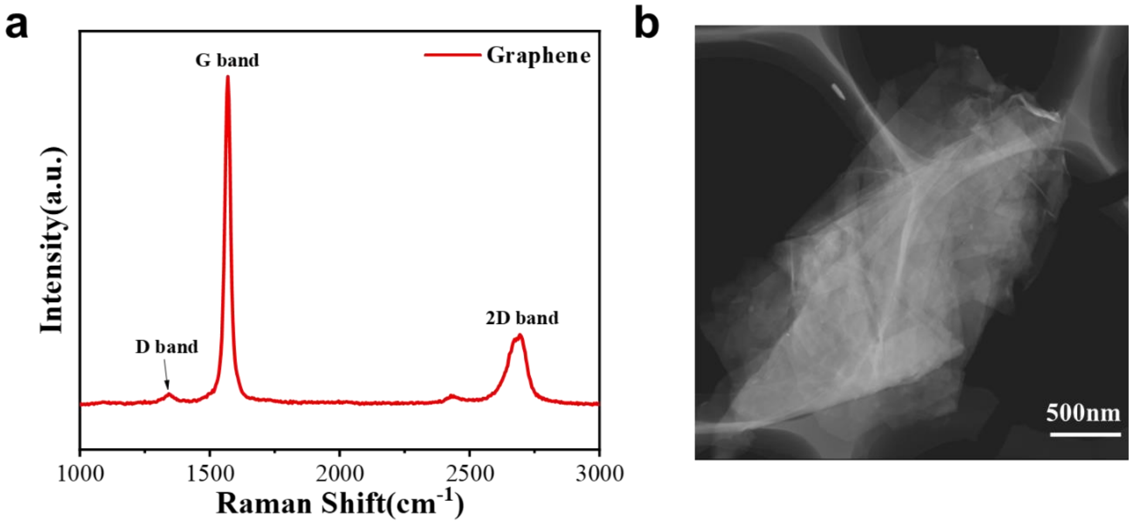

Supplementary Figure 5. a Raman spectrum of multilayer graphene; **b** TEM image of multilayer graphene.

The intensity ratio of D peak to G peak is usually used as an important parameter to characterize the defect density in the graphene. The I_D/I_G in Raman spectra follows the following equation:

$$\frac{I_D}{I_G} = C_A \frac{(r_A^2 - r_S^2)}{(r_A^2 - 2r_S^2)} \left[e^{-\pi r_S^2/L_D^2} - e^{-\pi(r_A^2 - r_S^2)/L_D^2} \right] \quad (1)$$

where r_S and r_A are the radii of the ‘structurally disordered’ area and the ‘activated’ area

surrounding the defect, respectively.[6] The factor C_A is defined by the electron-phonon matrix elements. Supplementary Reference. [7] reported that, if the wavelength of the excitation laser is in the visible light range, the mean distance between two adjacent defects (L_D, nm) in the graphene can be calculated using the following equation:

$$L_D^2(\text{nm}^2) = (1.8 \pm 0.5) \times 10^{-9} \lambda_L^4 \left(\frac{I_D}{I_G} \right)^{-1} \quad (2)$$

For the excitation laser with a wavelength of 532 nm (e.g., an energy of 2.33 eV), the mean distance between two adjacent defects (L_D, nm) in the graphene can be calculated from:

$$L_D(\text{nm}) = \sqrt[2]{1.26 \times 10^2 \left(\frac{I_D}{I_G} \right)^{-1}} \quad (3)$$

The defect density (n_D) can be calculated using $n_D(\text{cm}^{-2}) = 10^{14}/(\pi L_D^2)$, therefore,

$$n_D(\text{cm}^{-2}) = 2.58 \times 10^{11} \left(\frac{I_D}{I_G} \right) \quad (4)$$

As the relative intensity ratio (I_D/I_G) is about 0.615, the defect density n_D is about 1.5867×10^{11} (cm^{-2}).

In order to obtain the relevant information of the graphene flake, such as its average sheet size, atomic force microscope (AFM) was used. The obtained AFM images is shown in the Supplementary Figure 6. From the cross section line along the height shown in Supplementary Figure 6, we can obtain that the graphene flake has an average lateral size of ~1 μm and its thickness is about 1.5 nm.

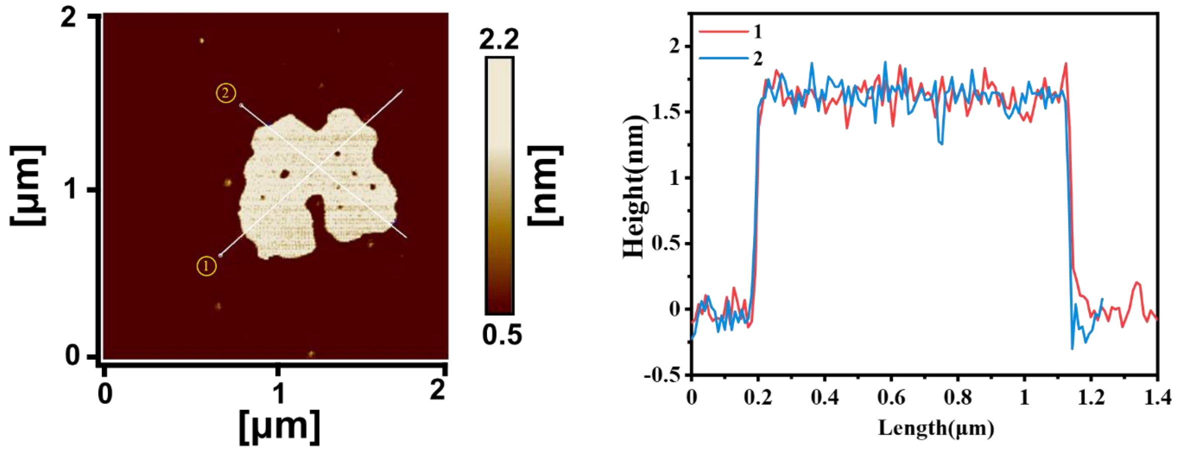

Supplementary Figure 6. AFM image of the graphene flake and corresponding height profile.

Supplementary Note 7. Surface morphology of Ecoflex B

The surface of Ecoflex B has many microscale wrinkles as shown in Supplementary Figure 7.

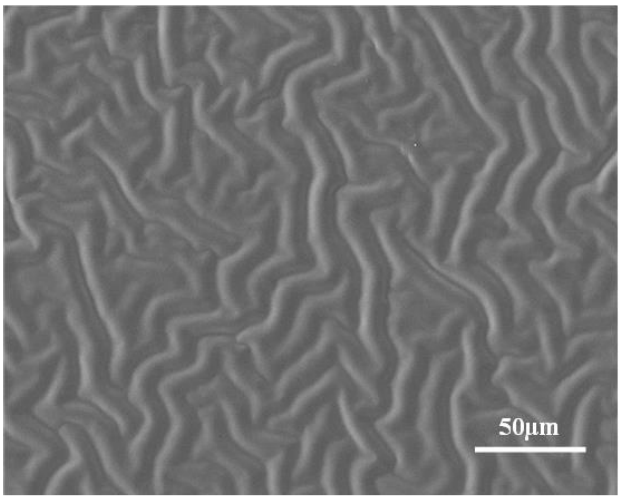

Supplementary Figure 7. SEM image of the surface morphology of Ecoflex B.

Supplementary Note 8. Comparisons of thickness for the graphene conductive layer of different samples

We have used the SEM to observe the cross-section morphologies of the graphene/Ecoflex sample at different magnifications. The results are shown in Supplementary Figure 8. They can provide the information of thickness estimation of graphene layer in this study.

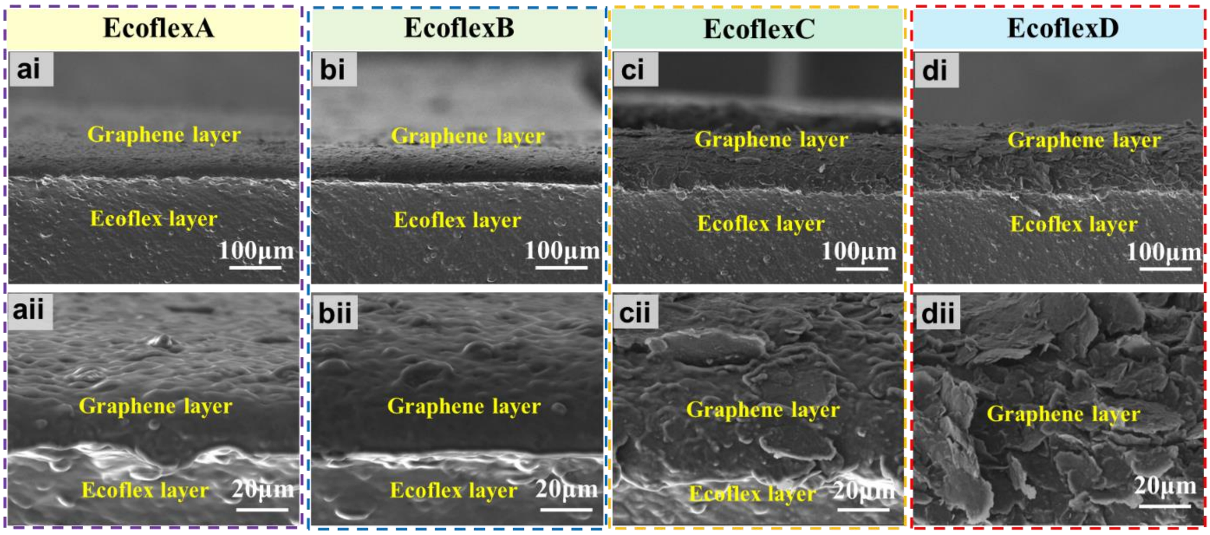

Supplementary Figure 8. The SEM images of the cross-sectional morphology of **ai~aii** graphene/Ecoflex A with different scale bar; **bi~bii** graphene/Ecoflex B with different scale bar; **ci~cii** graphene/Ecoflex C with different scale bar; **di~dii** graphene/Ecoflex D with different scale bar.

Supplementary Note 9. Relative resistance response of sensor at frequencies range from 0.35 Hz to 0.5 Hz

Supplementary Figure 9 shows the real-time frequency responses and output signals obtained from the strain sensor at different frequencies (from 0.35 Hz to 0.5 Hz) under a strain of 50%. Results reveal that the electrical responses of the sensor show good dynamic characteristics within the frequency range from 0.35 Hz to 0.5Hz. However, due to the speed limit of our tensile testing machine, the largest stretching frequency which can be used in the tests is 0.5 Hz.

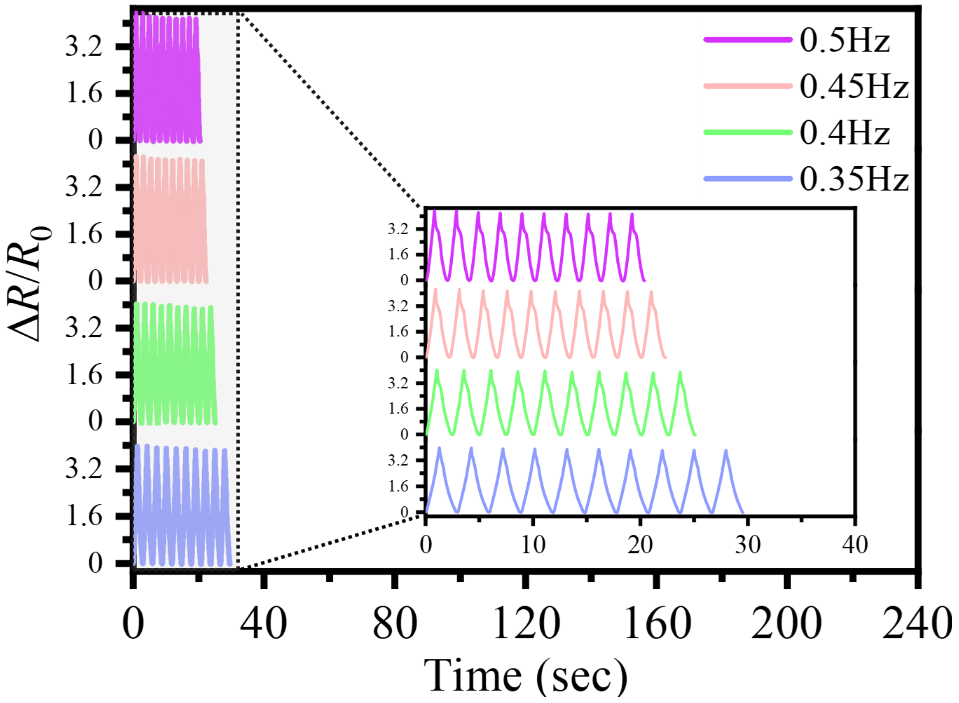

Supplementary Figure 9. Relative resistance response of the strain sensor at frequency from 0.35 to 0.5 Hz under a strain of 50%

Supplementary Note 10. Cycling durability of graphene/Ecoflex composite strain sensor under 300% strain

The graphene/Ecoflex composite strain sensor can be operated under a large strain of 300%, and it can guarantee 1500 cycles (an error less than 15%). The cycling durability curve is shown in Supplementary Figure 10.

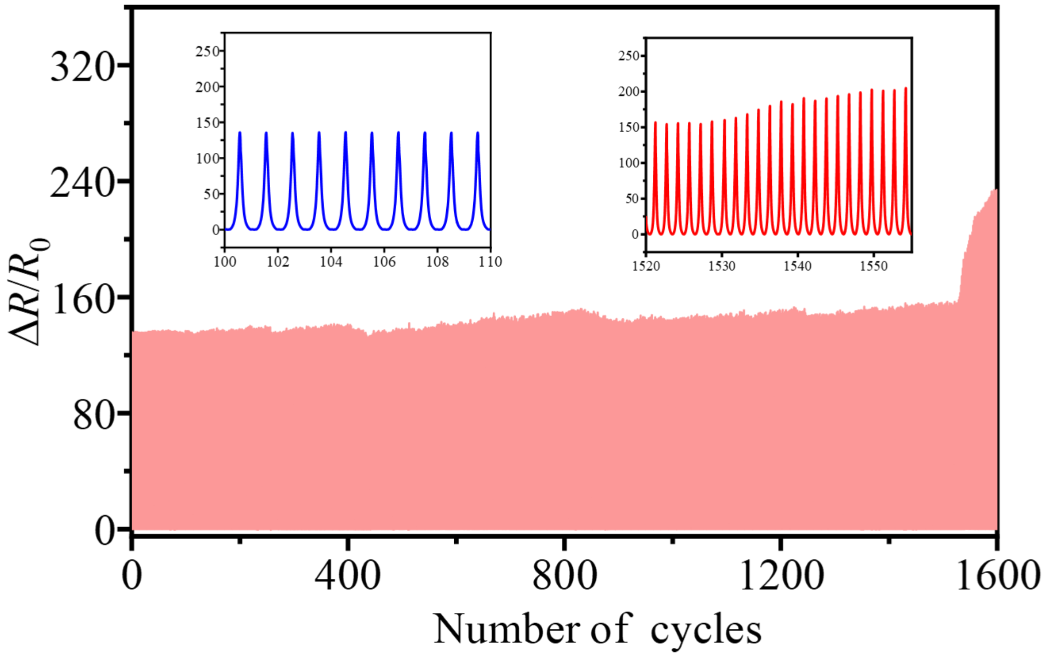

Supplementary Figure 10. The cycling durability curve of the strain sensor under 300% strain

Supplementary Note 11. Temperature effect experiments

We have built a simple test platform to test the effect of temperature on the performance of the sensor. Supplementary Figure 11 shows the test platform and the effect of temperature on the initial resistance. The obtained experimental data clearly show that temperature can slightly cause the changes of the resistance. It does not have an apparent influence on the conductivity of the sensor, compared with the effect of strain. The possible methods to minimize or eliminate such influence of temperature is to use a reference device or use a thermal isolation package .

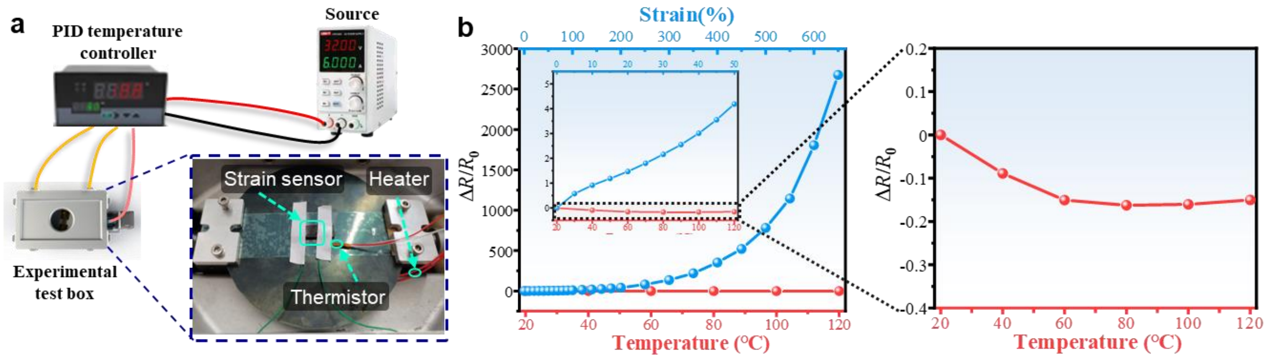

Supplementary Figure 11. Temperature effect experiments. **a** The test platform of investigation of the influence of temperature on the conductivity of the sensor; **b** the relative resistance changes ($\Delta R/R_0$) versus temperature and strain respectively.

Supplementary Note 12. Literature search and discussions on the pros and cons of those previously reported methods and our method.

Shi et al. reported a graphene-based resistance device with a fish-scale microstructure and achieved a sensing strain up to 82%, a gauge factor (GF) of 16.2~150, with excellent reliability and stability (>5000 cycles).[8] This fish-scale microstructure allows the neighboring overlapped graphene layers to readily change their overlapping areas via reversible slipping, and thus change their contact resistances. Accordingly, a high sensitivity and a low limit of detection have been achieved. However, this method involves multiple pre-stretching films and repeated pasting rGO films, which results in a complicated preparation process and is also prone to failures. Cai et al. proposed a strain sensor based on $Ti_3C_2T_x$ MXene/Carbon Nanotube composite, which was fabricated by repeatedly spraying MXene and CNT coating.[9] This kind of conductive layer takes the advantages of two materials thus achieving large sensitivity and detection range. However, the process of repeated spraying coating is also complicated. Amjadi et al. developed a resistance strain sensor based on reversible microcracks formed in a graphite composite thin film.[10] The sensor with these microsize cracks showed an ultrahigh sensitivity (with a maximum value of 11344) but a limited stretchability ($\varepsilon \leq 50\%$). Chen et al. also developed a strain sensor based a microcrack mechanism using an acid-interface engineering method.[11] The sensor showed a high sensitivity, but its detection range was very limited.

Kim et al. reported a strain sensor made of composite film with a densely packed microprism-array architecture. This sensor simultaneously achieved a good sensitivity (gauge factor≈81 at >130% strain) and a large stretchability (150%), as well as a long-term reliability (10000 cycles at a strain of 150%).[12] This densely packed microprism-array architecture leads to significantly morphological changes in the metal nanowires percolation network upon stretching. However, the equipment for the preparation of micropatterned silicon master design is relatively expensive, and the preparation process is complicated.

Different from all those previously reported methods of flexible strain sensors, we proposed a novel methodology by modulating multiscale and hierarchical wrinkles on the surface of a flexible substrate to be integrated into graphene/Ecoflex composite strain sensors.

The process mainly involves solution treatment method, which does not involve any expensive equipment and/or complicated preparation processes. Besides, by precisely controlling the solution concentration and treatment durations, high performance samples can be obtained (e.g., a large stretchability of up to 650% strain and a GF of up to 1078.1), with good process repeatability. Although the sensitivity of our sensor is slightly less than those based on microcrack mechanism, our sensor's detection range is much wider, which indicates that they can be used for a wide-range applications of both large and small strain scenario.

Of course, our sensor and its process still have some limitations. Firstly, compared with those based on a single step treatment, our two-step treatment method could increase the time of preparation process. In addition, our method is not IC compatible. Finally, the strain sensor shows obvious overshoots during its usage, which is due to the tensile stress-relaxation behavior under the applied strains.

Supplementary Note 13. Graphene/Ecoflex composite strain sensor applied for human motion detection

The wearable sensors were conformally attached to different positions of a person's body (for example, the elbow as shown in Supplementary Figure 12a) to monitor the person's motions. The sensor can correctly record and identify various motions of the elbow, showing the distinctly different patterns of response curves. We also attached the sensor at the throat to record the pressure signals. It can capture breathing and swallowing movements of the throat very well (Supplementary Figure 12b). For monitoring subtle physiological signals from the human body, we attached the wearable sensors to the wrist and recorded the wrist pulse under normal/exercise conditions. The obtained current signals are shown in Supplementary Figure 12c. The amplitudes and frequencies of pulses were recorded in real time (each peak denotes one pulse). Under the relaxation condition, the signal was calculated to be 66 beats/min, whereas after exercise, both the frequency and amplitude of the signals were significantly increased, with an increased pulse frequency of 84 beats/min.

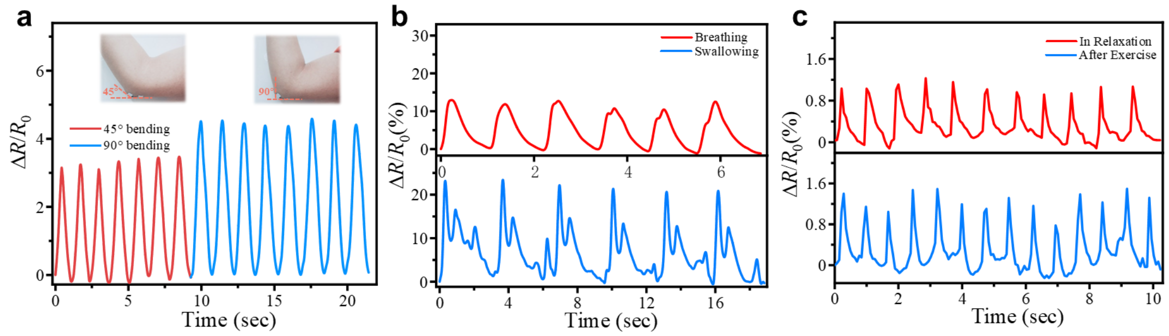

Supplementary Figure 12. Applications of graphene/Ecoflex composite strain sensor for monitoring various human motions. **a** Real-time resistance changes of elbow bending; **b** Breathing and swallowing movements of throat; **c** Physiological signals of wrist pulse.

Supplementary Note 14. Capability of the system for speech recognition with similar pronunciation

We have used similar sound/pronunciation to test the ability of the system for speech recognition, and the results are shown in Supplementary Figure 13. The phonetic sign of the word 'quite' is [kwaɪt], while the phonetic sign of the word 'quiet' is [ˈkwaɪət]. These two words are pronounced with a similar sound. 'quiet' has a stressed symbol in front of it. 'ə' is a vowel, which requires the vocal cords to vibrate when it occurs. 't' is a voiceless sound, which is produced by oral ventilation, and the vocal cords does not vibrate much. Based on the above reasons, the resistance changes of the vocal of 'quiet' have a small plateau in addition to the first peak compared to 'quite'.

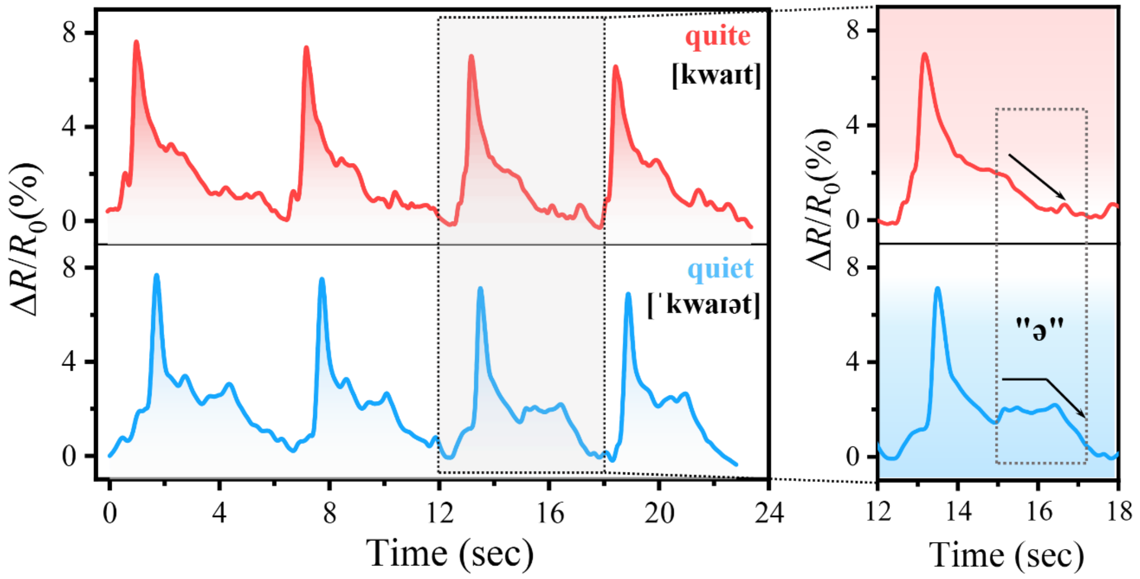

Supplementary Figure 13. Resistance changes in response to similarly sounding letters 'quite' and 'quiet'

Supplementary Reference

1. May, P., Khan, U., Hughes, J. M. & Coleman, J. N. Role of Solubility Parameters in Understanding the Steric Stabilization of Exfoliated Two-Dimensional Nanosheets by Adsorbed Polymers. *J. Phys. Chem. C* **116**, 11393-11400 (2012).

2. Hansen, C.M. Hansen Solubility Parameters: A User's Handbook. (CRC Press, 2007).

3. Boland, C. S. et al. Sensitive, High-Strain, High-Rate Bodily Motion Sensors Based on Graphene–Rubber Composites. *ACS Nano* **8**, 8819-8830 (2014).

4. Ferrari, A. C. et al. Raman Spectrum of Graphene and Graphene Layers. *Phys. Rev. Lett.* **97**, 187401 (2006).

5. Shi, M.-M. et al. Anchoring PdCu Amorphous Nanocluster on Graphene for Electrochemical Reduction of N2 to NH3 under Ambient Conditions in Aqueous Solution. *Adv. Energy Mater.* **8**, 1800124 (2018).

6. Cancado, L. G. et al. Quantifying defects in graphene via Raman spectroscopy at different excitation energies. *Nano Lett.* **11**, 3190-3196 (2011).

7. Lucchese, M. M. et al. Quantifying ion-induced defects and Raman relaxation length in graphene. *Carbon* **48**, 1592-1597 (2010).

8. Liu, Q., Chen, J., Li, Y. & Shi, G. High-Performance Strain Sensors with Fish-Scale-Like Graphene-Sensing Layers for Full-Range Detection of Human Motions. *ACS Nano* **10**, 7901-7906 (2016).

9. Cai, Y. et al. Stretchable Ti3C2Tx MXene/Carbon Nanotube Composite Based Strain Sensor with Ultrahigh Sensitivity and Tunable Sensing Range. *ACS Nano* **12**, 56-62 (2018).

10. Amjadi, M., Turan, M., Clementson, C. P. & Sitti, M. Parallel Microcracks-based Ultrasensitive and Highly Stretchable Strain Sensors. *ACS Appl. Mater. Interfaces* **8**, 5618-5626 (2016).

11. Chen, S. et al. Acid-Interface Engineering of Carbon Nanotube/Elastomers with Enhanced Sensitivity for Stretchable Strain Sensors. *ACS Appl. Mater. Interfaces* **10**, 37760-37766 (2018).

12. Kim, K.-H., Jang, N.-S., Ha, S.-H., Cho, J. H. & Kim, J.-M. Highly Sensitive and

Stretchable Resistive Strain Sensors Based on Microstructured Metal Nanowire/Elastomer Composite Films. *Small* **14**, 1704232 (2018).
